# Construction and validation of a prognostic signature based on seven endoplasmic reticulum stress-related lncRNAs for patients with head and neck squamous cell carcinoma

**DOI:** 10.1038/s41598-023-49987-1

**Published:** 2023-12-16

**Authors:** Mingzhu Zhou, Huihui Li, Juanjuan Hu, Tao Zhou, Liuqing Zhou, Yuncheng Li

**Affiliations:** 1 Department of Otorhinolaryngology, Union Hospital, Tongji Medical College, Huazhong University of Science and Technology, Wuhan, 430022 China; 2 Physical Examination Center, Union Hospital, Tongji Medical College, Huazhong University of Science and Technology, Wuhan, China

**Keywords:** Biomarkers, Head and neck cancer, Tumour biomarkers, Tumour immunology

## Abstract

Endoplasmic reticulum stress (ERS) occurs when misfolded or unfolded proteins accumulate in the endoplasmic reticulum (ER), and it is often observed in tumors, including head and neck squamous cell carcinoma (HNSCC). Relevant studies have demonstrated the prognostic significance of ERS-related long non-coding RNAs (lncRNAs) in various cancers. However, the relationship between ERS and lncRNAs in HNSCC has received limited attention in previous studies. In this study, we aimed to develop an ERS-related lncRNAs prognostic model using correlation analysis, Cox regression analysis, least absolute shrinkage, and selection operator (LASSO) regression analysis based on data from The Cancer Genome Atlas (TCGA) database. The survival and predictive ability of this model were evaluated using Kaplan–Meier analysis and time-dependent receiver operating characteristics (ROC), while nomograms and calibration curves were constructed. Then, functional enrichment analyses, tumor mutation burden (TMB), tumor infiltration of immune cells, single sample Gene Set Enrichment Analysis (ssGSEA), and drug sensitivity analysis were performed. Additionally, we conducted a consensus cluster analysis to compare differences between subtypes of tumors. Finally, we validated the expression of the ERS-related lncRNAs that constructed prognostic risk score model in HNSCC tissues through quantitative real-time PCR (qRT-PCR). We developed a prognostic signature based on seven ERS-related lncRNAs, which showed better predictive performance than other clinicopathological features. The high-risk poor prognosis group had a poorer prognosis in comparison to the low-risk good prognosis. The area under the ROC curve (AUC) predicted by this model for 3-year survival rates of HNSCC patients was 0.805. Enrichment analysis revealed that the differentially expressed genes were primarily enriched in pathways related to immune responses and signal transduction. Low-risk patients had lower TMB, more immune cell infiltrations, and enhanced anti-tumor immunity. Cluster analysis indicated that cluster 3 may have a better prognosis and immunotherapy effect. In addition, the result of qRT-PCR was consistent with our analysis. This prognostic model based on seven ERS-related lncRNAs is a promising tool for risk stratification, survival prediction, and immune cell infiltration status assessment.

## Introduction

Head and neck squamous cell carcinomas (HNSCC) are a group of aggressive, occult, and difficult-to-treat cancers that are estimated to have 840,000 cases in 2020 and are expected to rise to 1 million by 2030^1^. The modalities of therapy for HNSCC include surgical intervention, radiotherapy, and chemotherapy. However, these therapeutic approaches are often associated with loss of organ function and reduced quality of life. Moreover, they exhibit limited efficacy in managing advanced-stage patients^2–4^. In recent years, targeted immunotherapy has significantly improved the survival of patients with HNSCC. However, despite these advancements, approximately 60% of patients still have a poor response to these treatments^5^. Therefore, given the alarming surge in the incidence of HNSCC, it is imperative to identify efficacious prognostic biomarkers that can enhance the prognosis and treatment efficacy for patients with HNSCC.

Endoplasmic reticulum stress (ERS) is a homeostatic mechanism that refers to the excessive accumulation of unfolded or misfolded proteins in the lumen of the endoplasmic reticulum under the influence of stress factors such as hypoxia, oxidative stress, calcium depletion, and microenvironmental stress^6^. In response to ER stress, cells activate an adaptive signaling pathway known as the unfolded protein response (UPR) to cope with environmental changes and restore the ER to its normal state by regulating specific gene expression^7^. Multiple evidence suggests that ERS may regulate numerous aspects of tumor cell biology, such as angiogenesis, cell proliferation, tumor metabolism, and therapeutic resistance^8–10^. ERS plays a functional role in HNSCC by regulating key tumor biological processes including disease progression and treatment resistance^8^. Taken together, ERS may be a promising therapeutic target for the treatment of HNSCC.

Long non-coding RNA (lncRNA) is a type of non-protein-coding RNA (ncRNA) that exceeds 200 nucleotides in length and can participate in regulating protein-coding genes at various levels^11^. With unprecedented advances in understanding the function of lncRNAs, there is increasing evidence indicating that lncRNAs play a crucial role in various cancers^12^. Specifically, lncRNAs can be involved in cancer progression and metastasis^13,14^, closely related to the tumor microenvironment and therapeutic resistance^15^, and have great potential as prognostic or diagnostic biomarkers for tumors^16^. A growing number of lncRNAs are involved in the occurrence and progression of HNSCC, which can predict patients' prognosis and become potential therapeutic targets^17^. However, the prognostic potential of ERS-related lncRNAs in HNSCC remains largely unexplored. In this study, we developed a prognostic signature based on seven ERS-related lncRNAs for patients with HNSCC. Subsequently, we investigated the impact of these lncRNAs on HNSCC prognosis.

## Materials and methods

**Figure 1:**
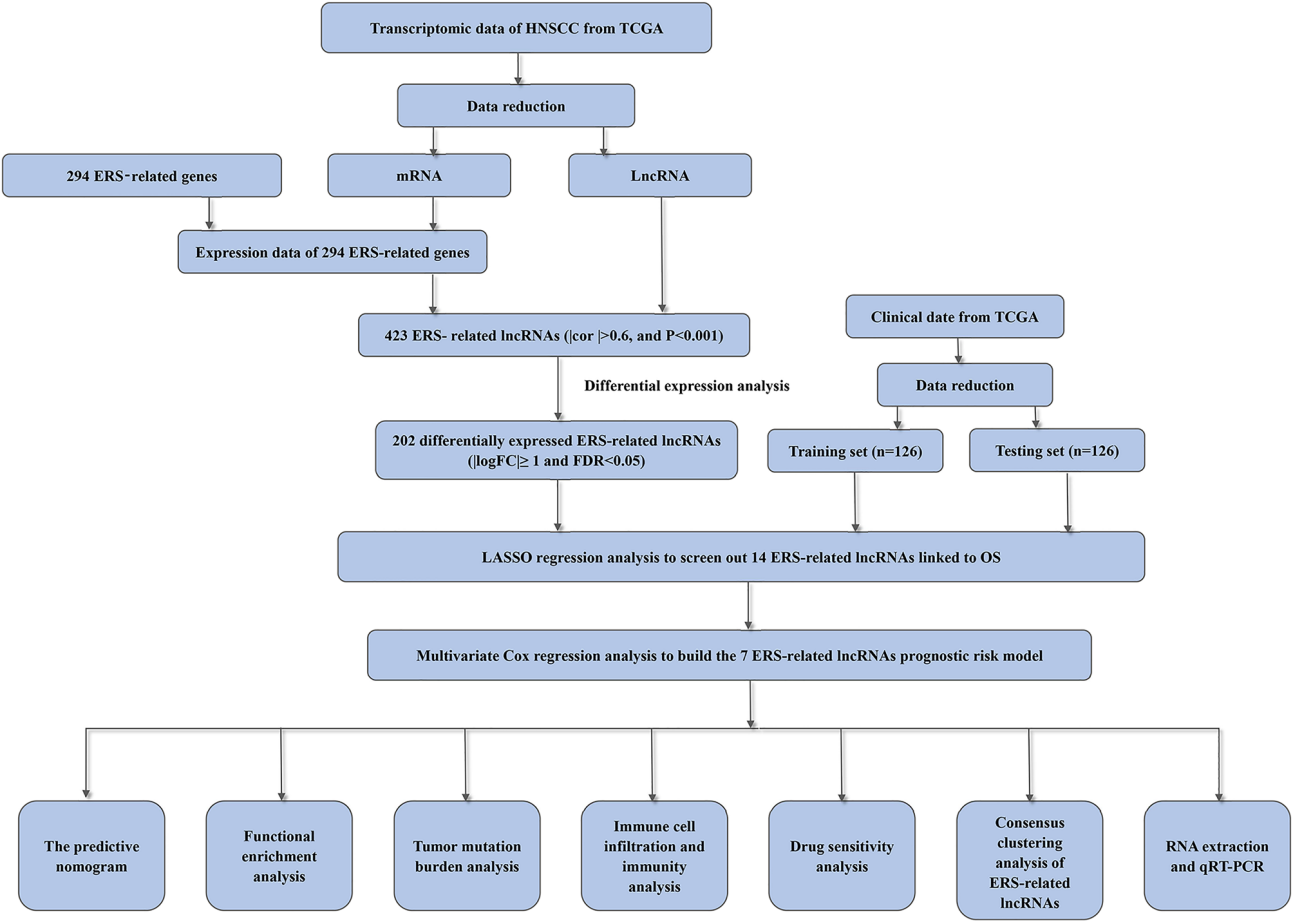
The flow chart of this study.

The flow chart of this study is illustrated in Fig. 1.

### Data collection

RNA-sequence (17 normal and 254 tumors) data and corresponding clinical information of 271 patients were extracted from the TCGA-HNSCC database (https://portal.gdc.cancer.gov/). Then, lncRNA data were collated and screened from RNA-sequence data for the subsequent difference and co-expression analysis. We acquired the somatic mutation data of the HNSCC samples from the TCGA GDC Data Portal in ‘maf’ format.

### Identification of ERS‑related lncRNAs

A total of 294 ERS‑related genes were identified from published studies. Then, the "limma" R package was utilized to construct ERS-related gene expression matrices and identify ERS‑related lncRNAs in R software (version 4.0.5)^18^. To identify ERS‑related lncRNAs, the selection criteria included a correlation coefficient |cor|> 0.6 and *p* < 0.001. Subsequently, we used the R packages "limma" and "pheatmap" to identify differentially expressed ERS-related lncRNAs between normal samples and HNSCC samples, with a cutoff of |log2 fold change (FC)|≥ 1.0 and false discovery rate (FDR) < 0.05.

### Construction and validation of the ERS-related lncRNAs prognostic signature

To improve the credibility of the risk score, we divided all HNSCC cases into training and test sets in a 1:1 ratio. The training set was employed for developing a prognosis prediction model, while the testing set was utilized to assess the prediction performance. To begin with, univariate Cox regression analysis was applied to determine the relationship between differentially expressed ERS-related lncRNAs and the prognosis of HNSCC patients. Forest map and heatmap were plotted, and *p* < 0.05 was used as the threshold to identify differentially expressed ERS-related lncRNAs. Later, to reduce the overfitting of the ERS-related prognostic lncRNAs, least absolute shrinkage and selection operator (LASSO) regression analysis were utilized to further screen the above lncRNAs. Afterward, multivariate Cox regression analysis was used to identify independent prognostic indicators for model construction based on the Akaike information criterion (AIC) value^19^. The prognostic risk score was calculated as follows: $Risk score =\sum_{K=1}^{7}X\left(k\right) \times Coef(k)$, X (k), and Coef (k) represents the corresponding expression level of ERS-related lncRNAs and the risk coefficient derived from the multivariate regression analysis, respectively. Finally, the patients were divided into a high-risk poor prognosis group and a low-risk good prognosis group based on their median risk scores. The high-risk poor prognosis group was defined as those with risk scores surpassing the median value, indicating an unfavorable prognosis. Conversely, the low-risk good prognosis group comprised individuals with risk scores below the median value, suggesting a more favorable prognosis.

To validate the prognostic ability of the developed model, a range of analyses were conducted. Kaplan–Meier analysis was done for inter-group overall survival. Time-dependent ROC curve analysis was used to evaluate the prediction accuracy of the constructed risk prediction model. In addition, univariate and multivariate Cox regression analyses were applied to verify the independent prognostic ability of the developed model. The "survival", "survminer", and "timeROC" R packages were used for the aforementioned analyses.

### The predictive nomogram

A nomogram was constructed using the "rms" R package to predict the overall survival (OS) of HNSCC patients at 1, 2, and 3 years^20^. The calibration curves and concordance index (C-index) were utilized to assess the predictive ability of the nomogram^21^. When the C-index was higher than 0.7, the model could be considered to have good predictive performance.

### Functional enrichment analysis

The "limma" R package was used to screen for genes that were differentially expressed between the high-risk poor prognosis and low-risk good prognosis groups (|logFC|> 1 and FDR < 0.05). The Gene Ontology (GO) and Kyoto Encyclopedia of Genes and Genomes (KEGG) analyses were conducted to identify the functions and biological pathways linked to the ERS-related lncRNAs. Additionally, we conducted Gene Set Enrichment Analysis (GSEA) to investigate the potential signaling pathways in both groups. Gene sets with a *p* < 0.05 were considered significantly enriched.

### Tumor mutation burden (TMB) analysis

Tumor mutational burden (TMB) is a quantification of genomic instability in cancer cells, representing the cumulative count of somatic mutations identified per million bases. Research has indicated that patients with a high TMB are more likely to benefit from immunotherapy^22,23^. The "Maftools" package in R software was used to generate waterfall plots, which visualize the mutated genes in both high-risk poor prognosis and low-risk good prognosis groups. In addition, the Kaplan–Meier curves were performed using "survival" and "survminer" packages to investigate the impact of risk score and TMB on patient survival.

### Immune cell infiltration and immunity analysis

The correlation between immune cells and risk scores was explored using various algorithms, including XCELL^24^, TIMER^25^, QUANTISEQ^26^, MCPCOUNTER^27^, EPIC^28^, CIBERSORT-ABS^29^, and CIBERSORT^29^. We further conducted Kaplan–Meier survival analyses to investigate the association between immune cells and the survival rates of patients with HNSCC. In addition, ssGSEA was used to quantify the subgroups of tumor-infiltrating immune cells between the high- and low-risk groups, as well as assess their immune-related functions. The Wilcoxon test was used to analyze the differential expression of immune checkpoint genes between the two groups, and the results were presented in a boxplot^30^.

### Drug sensitivity analysis

To assess the predictive ability of our model for treatment response in HNSCC, we obtained the anticancer drug dataset from the Genomics of Drug Sensitivity in Cancer (GDSC) website (GDSC; https://www.cancerrxgene.org/). We then used the "oncoPredict" package to calculate the relationship between different risk groups and IC50 values for various anticancer drugs^31^. The Wilcoxon signed-rank test was utilized to compare the difference in IC50 between the high-risk poor prognosis and low-risk good prognosis groups, and the results were presented as box plots obtained using the "ggpubr" and "ggplot2" packages in R.

### Consensus clustering analysis of ERS-related lncRNAs

Consensus cluster analysis of the expression matrix of 7 ERS-related lncRNAs was performed using the "ConsensusClusterPlus" R package, resulting in the classification of HNSCC patients in the TCGA cohort into three clusters. The "survival" and "survminer" R packages were also used to compare the survival differences among tumor subtypes. Then, the relationship between sample subgroups and patient risk was presented using Sankey plots. The accuracy of classification based on the expression profiles of the aforementioned lncRNAs was verified through principal component analysis (PCA) and t-distributed stochastic neighbor embedding (t-SNE)^32^, using "ggplot2" and "Rtsne" R packages. Furthermore, we evaluated differences in tumor immune cell infiltration, immune checkpoints, and drug susceptibility among different clusters.

### RNA extraction and qRT-PCR

Tumor tissues and adjacent tissues were collected from eight HNSCC patients who underwent surgical resection at Wuhan Union Hospital and stored in − 80 °C refrigerator. All patients have signed informed consent forms prior to surgery. Total RNA was extracted from tissues using Trizol (Takara, China) and reversed to cDNA by the Prime Script RTase (Takara, China). Subsequently, SYBR green (Takara, China) was used for qRT-PCR. The internal control β-Actin was utilized to standardize the expression levels of lncRNAs. The primer sequences are shown in Table 1.

**Table 1:** The list of the primers used for qRT-PCR.

ID	Forward or reverse primer	The primer sequence (5′–3′)
ACTN1-AS1	Forward	AAAGGACGCGAGGAAACCC
	Reverse	CGAGGGCGAGCTAGAAGAGT
AP003774.2	Forward	GAACCAGGAAGCATTCTGAGGAG
	Reverse	AGTCATTTCTCAGTTCTGCCATCAC
DOCK8-AS1	Forward	GAATAATGAAGCAGGCGAGGAC
	Reverse	CACGGAGTGTCTCATAAACGGC
DDX11-AS1	Forward	ACCTCACCTCCCTAGACTTTGCT
	Reverse	GAAAGGTTGCTGGCTGATGGT
MIR924HG	Forward	CCTCTGGGCATGGGTACAAG
	Reverse	TCTCCGCTGAACTTTTGCCA
MIR9-3HG	Forward	CAGCAGGAAGGGAGAACAAGTA
	Reverse	ATGCACCTCAGACCCAAACA
AL451085.2	Forward	CAAAAGGAGGCCAGGATGAA
	Reverse	GCCCGAATGCCTGTCAGTTT

### Statistical analysis

Statistical analysis was performed using R software (version 4.0.5), and a *p*-value < 0.05 was considered statistically significant.

## Results

### Identification of ERS-related differentially expressed lncRNAs in HNSCC

**Figure 2:**
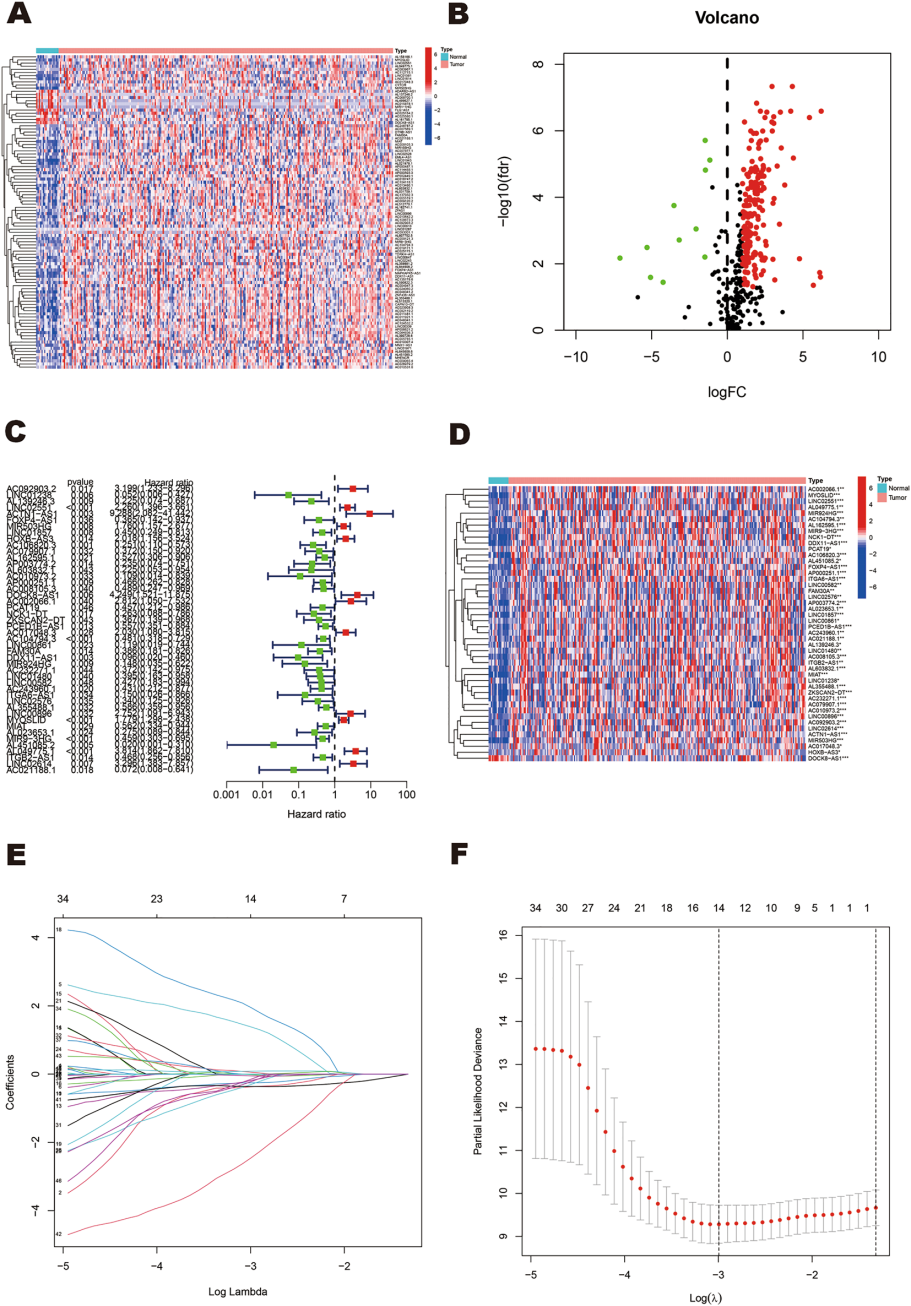
Identification of differentially expressed ERS-related lncRNAs and construction of an ERS-related lncRNAs model for HNSCC patients. (**A**) Heatmap of 100 significant differentially expressed lncRNAs. (**B**) Volcano plot of ERS-related lncRNAs. Red dots indicate upregulated lncRNAs in tumor tissues while the green dots indicate downregulated lncRNAs. Black dots indicate no significant difference in lncRNA expression between tumor and normal samples. (**C,D**) Forest plot (**C**) and heatmap (**D**) of the prognostic ERS-related lncRNAs extracted by the univariate Cox regression analysis. (**E,F**) LASSO regression analysis identified 14 lncRNAs most associated with OS in the TCGA dataset.

Based on the TCGA database, we obtained the RNASeq data and corresponding clinical data of 271 HNSCC patients. 423 ERS-related lncRNAs were obtained through co-expression analysis with 294 ERS‑related genes from published studies. Through differential analysis, we identified 202 ERS-related lncRNAs that showed significant differential expression between normal and tumor samples (|logFC|≥ 1 and FDR < 0.05). The heatmap presented the differential expression of 100 ERS-related lncRNAs between normal and HNSCC tissues (Fig. 2A). Among the 202 ERS-related differentially expressed lncRNAs, 191 lncRNAs were up-regulated in tumors while 11 lncRNAs were down-regulated. The result was shown in the volcano plot (Fig. 2B).

### Acquisition of ERS-related lncRNAs related to HNSCC prognosis

To identify ERS-related lncRNAs associated with HNSCC prognosis, we first collated the survival data of HNSCC tumor samples, removed samples without survival information, and retained 252 tumor samples for subsequent analysis. We then randomly divided the aforementioned samples into a training set and a testing set, with an approximate ratio of 1:1. Using univariate Cox regression analysis, we screened 46 ERS-related lncRNAs that were significantly associated with the prognosis of HNSCC patients in the training set (Fig. 2C). The heatmap displayed the expression of 46 ERS-related lncRNAs associated with prognosis in both normal and tumor tissues (Fig. 2D).

### Construction and validation of a prognostic signature

Based on the aforementioned 46 ERS-related lncRNAs screened from the training set, we performed LASSO regression analysis to further identify 14 ERS-related lncRNAs. The cvfit curve and lambda curve are shown in Fig. 2E, F. Importantly, seven prognostic ERS-related lncRNAs (ACTN1-AS1, AP003774.2, DOCK8-AS1, DDX11-AS1, MIR924HG, MIR9-3HG, and AL451085.2) were identified by multivariate Cox regression analysis and successfully used to build the prognostic risk model. In this model, the risk score was computed based on the coefficients of the aforementioned seven lncRNAs. The risk score = ACTN1-AS1 × (2.525895) + AP003774.2 × (−1.158778) + DOCK8-AS1 × (3.480702) + DDX11-AS1 × (−1.762455) + MIR924HG × (−1.710509) + MIR9-3HG × (−0.376870) + AL451085.2 × (−5.600491).

**Figure 3:**
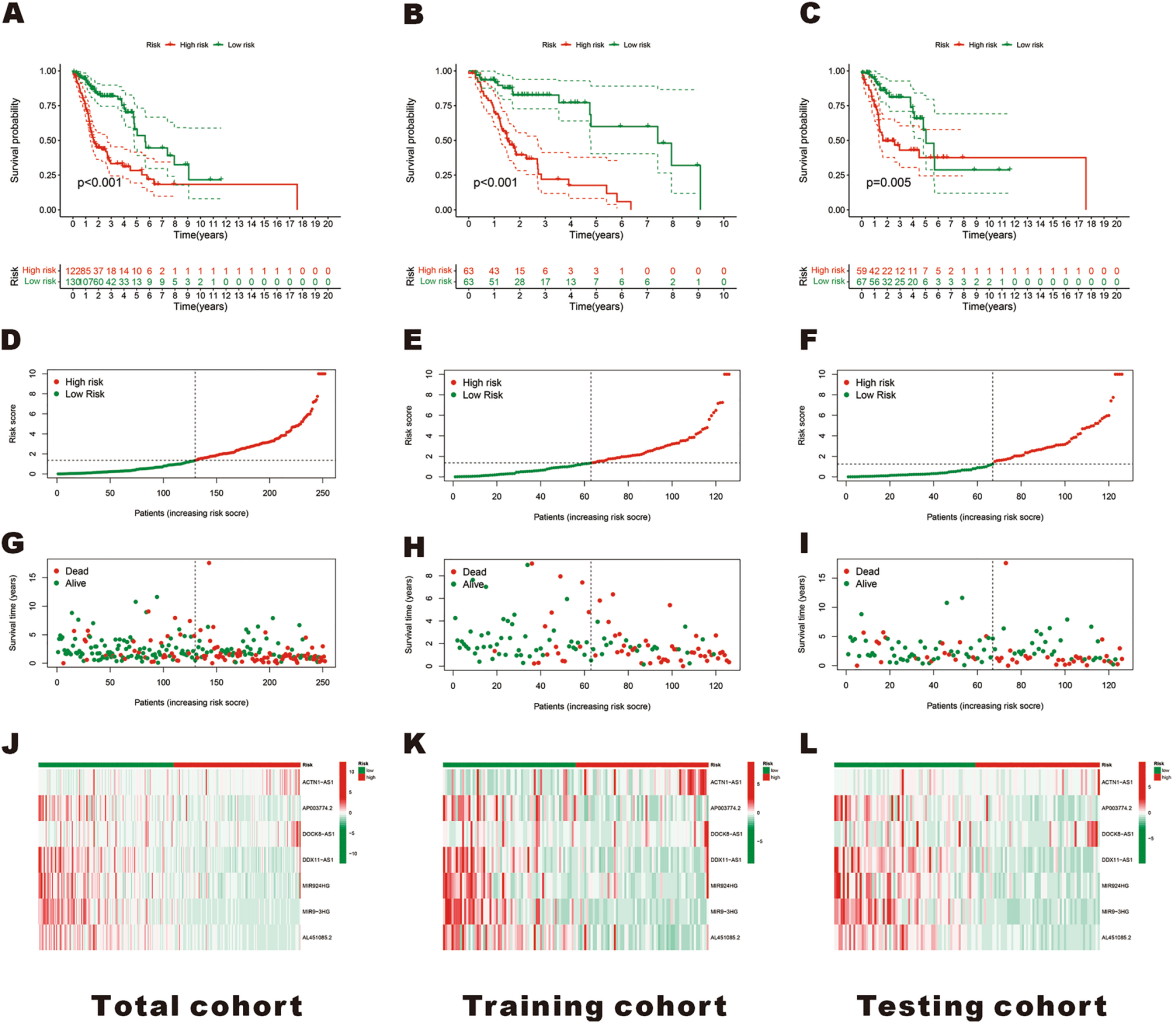
Construction and validation of the ERS-related lncRNAs signature model in the training, testing, and total cohorts. (**A–C**) Kaplan–Meier survival curves of HNSCC patients’ OS in the high-risk poor prognosis and low-risk good prognosis groups in the total (**A**), training (**B**), and testing cohorts (**C**). (**D–F**) Risk score distribution in the total (**D**), training (**E**), and testing cohorts (**F**) for two groups. (**G–I**) survival status distribution in the total (**G**), training (**H**), and testing cohorts (**I**). (**J–L**) Risk heatmaps of the 7 ERS-related lncRNA expression in the total (**J**), training (**K**), and testing cohorts (**L**).

To better verify the predictive accuracy of this model, HNSCC patients in each cohort were divided into high-risk poor prognosis and low-risk good prognosis groups based on their median scores in the training cohort. The Kaplan–Meier analysis was performed to assess the prognostic efficacy of this model for HNSCC patient's OS status, revealing that the survival rate of HNSCC patients in the low-risk good prognosis group was significantly higher than that in the high-risk poor prognosis group in the training, testing, and total cohorts (*p* < 0.01) (Fig. 3A-C). Based on the risk score and survival data of the patients in the different cohorts, the patient's risk score distribution map (Fig. 3D-F) and survival status were obtained, and it was observed that there was a positive correlation between the risk score and mortality rate among patients (Fig. 3G-I). Heatmaps revealed differences in the expression of seven ERS-related lncRNAs between high-risk poor prognosis and low-risk good prognosis groups across the training, testing, and total cohorts. From Fig. 3J-L, we know that the expression tendency of each lncRNAs is the same in different groups.

**Figure 4:**
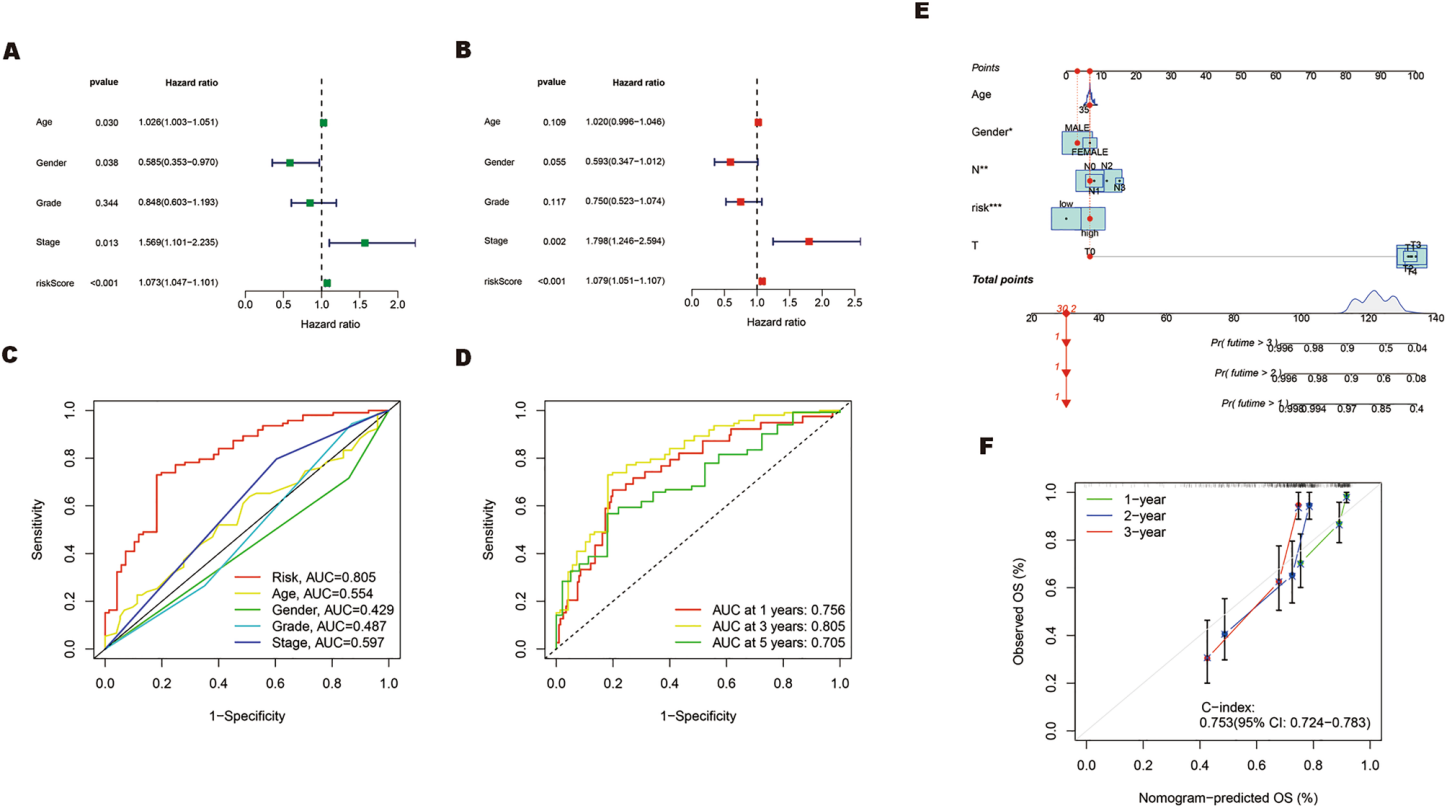
Evaluation of the prognostic value and construction of nomogram models for HNSCC. (**A,B**) Univariate (**A**) and multivariate (**B**) Cox analyses evaluate the independent prognostic value of ERS-related lncRNAs risk signature in HNSCC patients. (**C**) ROC curves and AUCs of the risk score and clinicopathological characteristics. (**D**) ROC curves and AUCs for 1-, 3-, and 5-year survival rates of the total cohort. (**E**) Nomogram plot was built based on risk and stage in the total cohort. (**F**) Calibration curve of the nomogram.

Next, univariate and multivariate Cox regression analyses were performed to assess the independent prognostic value of the risk score in patients with HNSCC. As shown in Fig. 4A, B, compared with other clinical factors, the risk score of this model was not only associated with the prognosis of HNSCC patients (HR = 1.073, 95% CI: 1.047–1.101, *p* < 0.001) but it could also effectively predict the survival of HNSCC patients independent of other clinical factors (HR = 1.079, 95% CI: 1.051–1.107, *p* < 0.001). ROC curve analysis was performed to analyze the sensitivity and specificity of this model. The results showed that the area under the ROC curves (AUC) of this model was 0.805, which was significantly larger than that of other factors, such as age (AUC = 0.554), gender (AUC = 0.429), grade (AUC = 0.487) and stage (AUC = 0.597) (Fig. 4C). In addition, the area under the ROC curve (AUC) predicted by this model for 1 -, 3- and 5-year survival rates of HNSCC patients was 0.756, 0.805, and 0.705, respectively (Fig. 4D), indicating the high accuracy of this model. All of these findings demonstrate the robustness of our model in predicting HNSCC survival.

### ERS-related lncRNA prognostic nomogram development and evaluation

To facilitate the prognostic evaluation of HNSCC patients in clinical practice, we have developed a nomogram based on clinical factors and risk scores. The nomogram was used to estimate the survival probability of patients with HNSCC at 1-, 2-, and 3- year (Fig. 4E). The calibration curves demonstrated consistency between predicted and actual observed 1-, 2-, and 3-year survival rates in HNSCC patients depending on the prognostic nomogram. Additionally, the C-index of 0.753 (95% confidence interval (CI), 0.724–0.783) surpassed the threshold of 0.7, indicating a good predictive capacity of this model (Fig. 4F)^33–35^.

### The functional and biological pathway analyses

**Figure 5:**
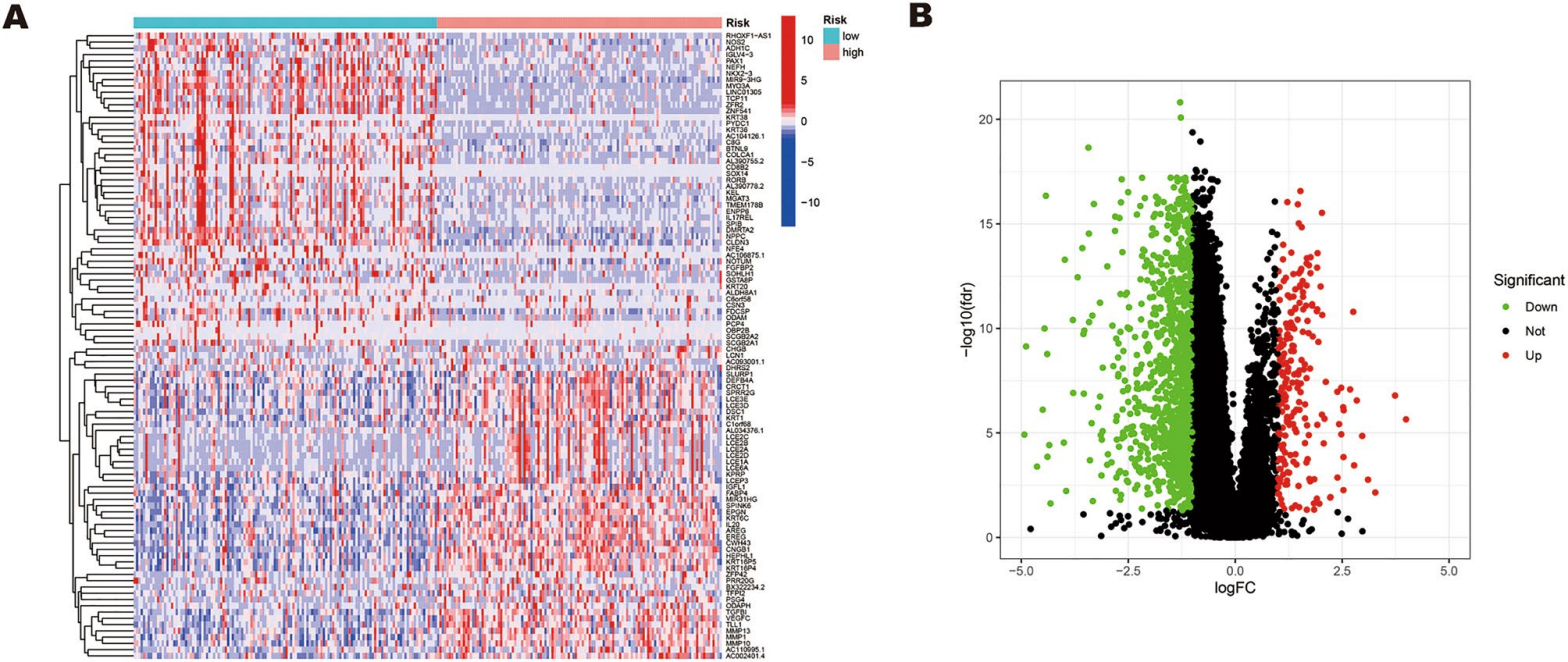
Identification of differentially expressed ERS-related genes in different risk groups. (**A**) Heatmap shows the expression of differentially expressed genes in different risk groups. Red represents high expression and blue represents low expression. (**B**) Volcano plot of differentially expressed genes in high-risk poor prognosis and low-risk good prognosis groups, red for high expression and green for low expression.

**Figure 6:**
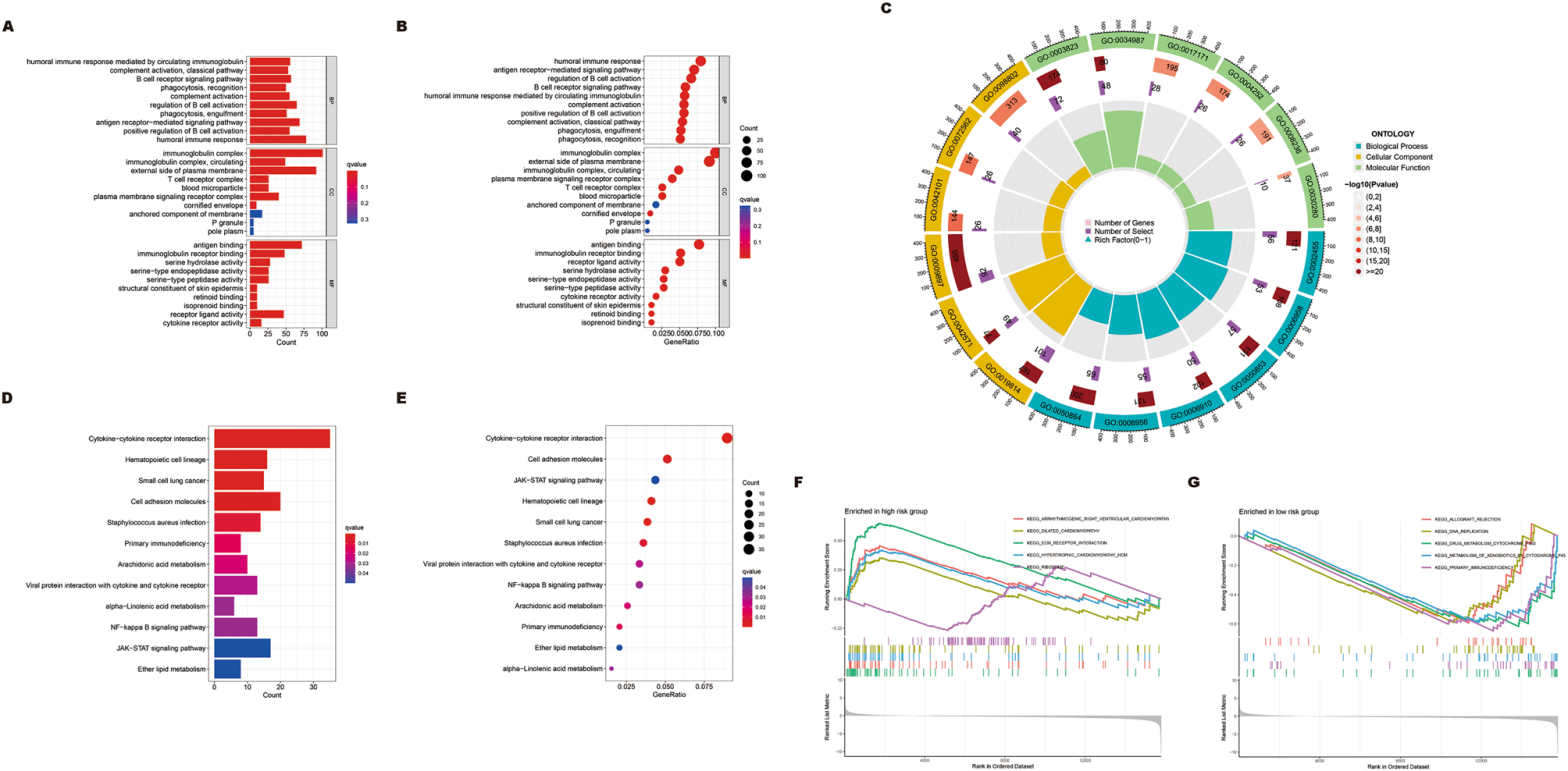
Functional enrichment analysis. (**A–C**) The GO function enrichment analyses. (**D,E**) The KEGG signaling pathway enrichment analysis. (**F,G**) GSEA analysis determines the underlying signal pathway between high-risk poor prognosis and low-risk good prognosis groups.

The expression differences and levels of gene expression in the high-risk poor prognosis and low-risk good prognosis groups were presented by heatmap (Fig. 5A) and volcano plot (Fig. 5B). GO and KEGG enrichment analyses were conducted on the aforementioned differentially expressed genes (DEGs) to investigate their potential functions and enriched pathways. As shown in Fig. 6A–C, these DEGs were mainly enriched in humoral immune response, antigen receptor-mediated signaling pathway, regulation of B cell activation, immunoglobulin complex, and antigen binding. KEGG enrichment analysis showed these DEGs were mainly enriched in cytokine−cytokine receptor interaction, cell adhesion molecules, and JAK−STAT signaling pathway (Fig. 6D, E). It can be seen that ERS may promote the growth and proliferation of cancer cells through the activation of the aforementioned pathways, which provides an idea to explore its potential mechanisms that promote cancer. To explore differential biological functions and pathways between the high-risk poor prognosis and low-risk good prognosis groups, we further conducted the GSEA enrichment analysis. Further GSEA analysis showed that immune-related pathways were enriched in low-risk good prognosis group, such as allograft rejection, and primary immunodeficiency. However, the high-risk poor prognosis group did not exhibit any notable enrichment of immune-related pathways (Fig. 6F, G), which may indicate a close association between ERS and tumor immunity.

### Tumor mutation burden (TMB)

**Figure 7:**
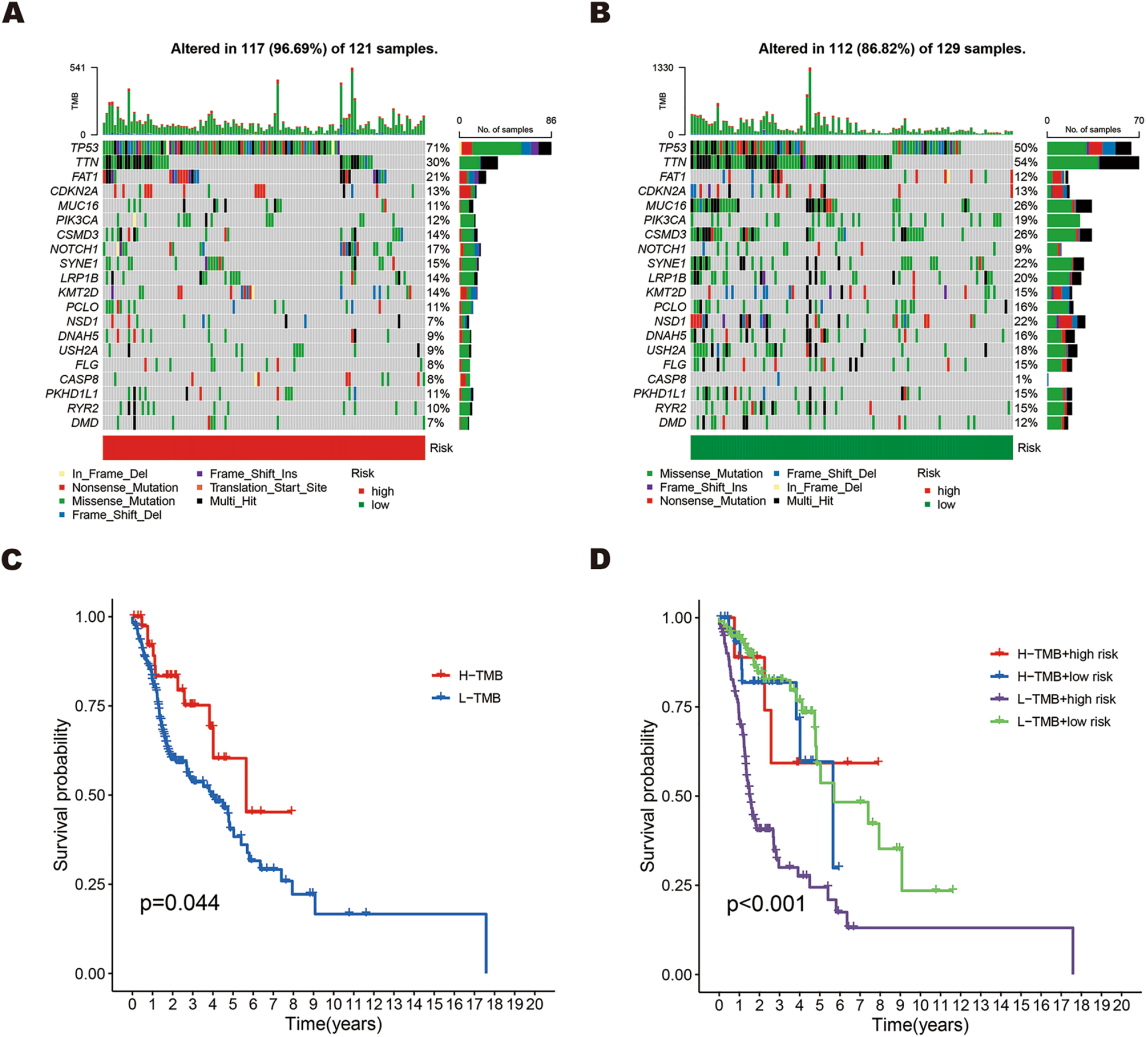
TMB analysis of the prognostic signature. (**A,B**) The waterfall plots of the tumor mutation rate in the high-risk poor prognosis (**A**) and low-risk good prognosis (**B**) based on the prognostic signature. (**C**) Kaplan–Meier survival curves of HNSCC patients between the H-TMB and L-TMB groups. (**D**) Kaplan–Meier survival curves of HNSCC patients across H-TMB + high risk, H-TMB + low risk, L-TMB + high risk, and L-TMB + low risk.

Currently, an increasing number of studies indicate that TMB has the potential to serve as a biomarker for immunotherapy and guide the clinical application of immune checkpoint inhibitors (ICIs)^22,23^. To better predict patient prognosis and treatment effects, we performed a TMB analysis and visualized the mutation status in high-risk poor prognosis and low-risk good prognosis groups. The differences in mutated genes and mutation rates between high-risk poor prognosis patients and low-risk good prognosis patients are illustrated through waterfall plots (Fig. 7A, B). As shown in the waterfall plots, the mutation rate of the high-risk poor prognosis group is significantly higher than that of the low-risk good prognosis group (96.69% vs. 86.82%). TP53 (71% vs. 50%) and TTN (30% vs. 54%) were identified as the main mutated genes, with different mutation rates observed between the high-risk poor prognosis and low-risk good prognosis groups. Based on the Kaplan–Meier survival curves of TMB, we observed that the high-mutation group had a better prognosis than the low-mutation group (Fig. 7C, p < 0.05). In addition, patients with low-TMB scores and high-risk scores had the poorest prognosis compared to the other groups, as shown in Fig. 7D. Based on these findings, it can be observed that patients with a high TMB have a more favorable prognosis and are more likely to benefit from ICIs.

### Immune cell infiltration analysis and immunotherapy efficacy evaluation of the ERS-related lncRNA prognostic signature

**Figure 8:**
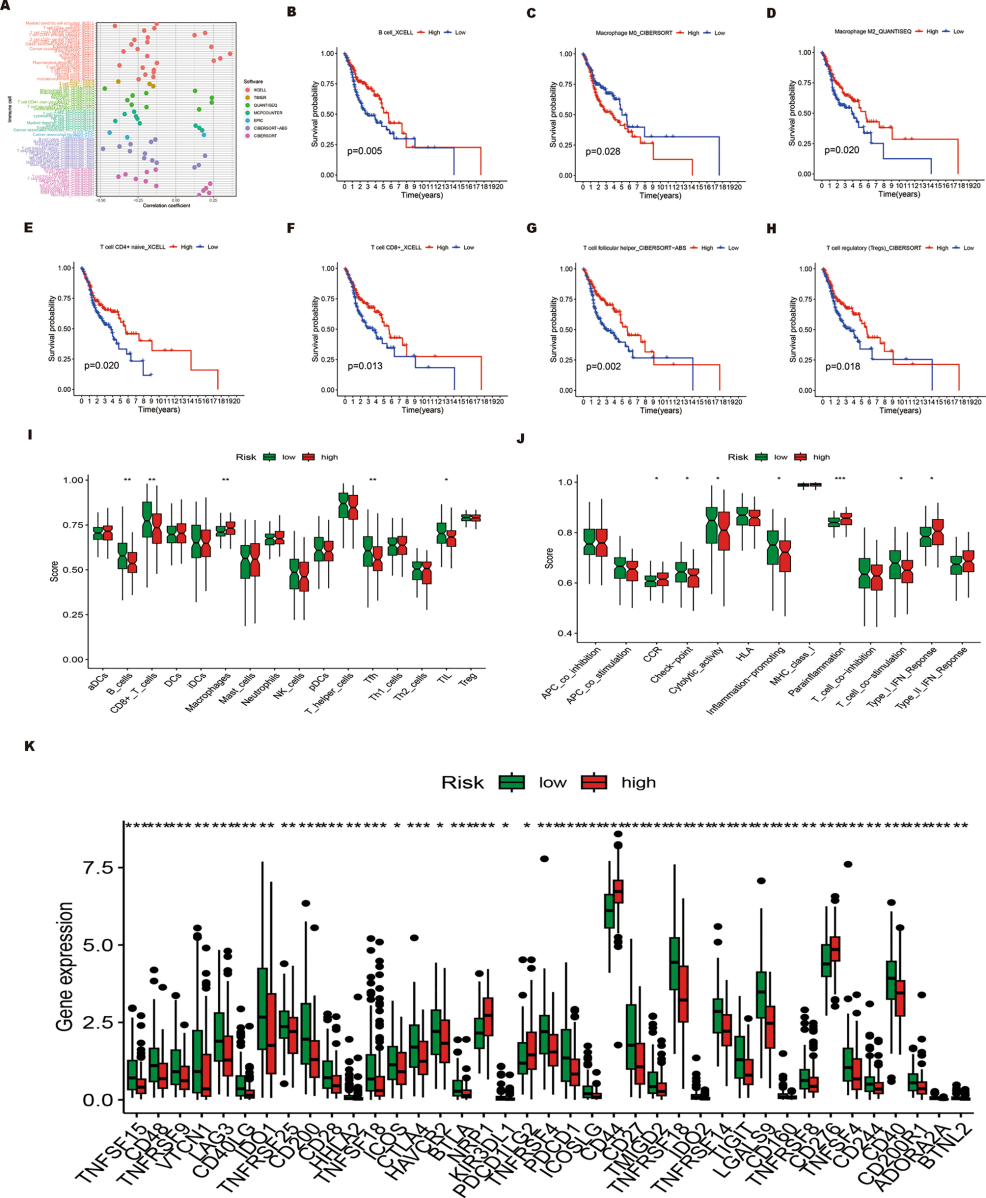
Exploration of the immune cell infiltration landscape in HNSCC patients. (**A**) Estimation of immune-infiltrating cells in HNSCC by using the Spearman correlation analysis. (**B–H**) Kaplan–Meier survival curves of B cells (**B**), macrophages M0 (**C**), macrophages M2 (**D**), CD4+ naïve T cells (**E**), CD8+ T cells (**F**), T follicular helper cells (**G**), and regulatory T cells (Tregs) (**H**) in HNSCC patients. (**I,J**) The score of the infiltrating immune cells (**I**) and immune-related functions (**J**) in the high-risk poor prognosis and low-risk good prognosis groups. (**K**) The expression of immune checkpoint-related genes in the high-risk poor prognosis and low-risk good prognosis groups.

Increasing evidence suggests that ERS may hinder the development of effective anti-cancer immunity^36^. While investigating the correlation between immune cell infiltration and risk score, our results demonstrate a significant inverse association between risk scores and the majority of infiltrating immune cells, including T cell CD8+ , B cell, T cell regulatory (Tregs), and macrophage M2, etc. However, some infiltrating immune cells were found to have a significant positive correlation with the risk score, including neutrophil, macrophage M1, mast cell resting, and NK cell resting (Fig. 8A, p < 0.05). Next, survival analysis was conducted to determine whether there were differences in OS between high- and low-expression groups of immune cells. The prognosis was worse for macrophage M0 in the high expression group compared to the low expression group, while other immune cells with high expression predicted a better prognosis, such as B cell, macrophage M2, T cell CD4+ naive, T cell CD8+, T cell follicular helper, and T cell regulatory T cells (Tregs) (Fig. 8B–H, p < 0.05). Considering the current increasing demand for personalized immunotherapy, we further used ssGSEA to quantitatively analyze immune cells and their related functions in both high-risk poor prognosis and low-risk good prognosis groups. The ssGSEA scores of B cells, CD8+ T cells, Tfh cells, and tumor-infiltrating lymphocytes (TILs) were significantly higher in the low-risk good prognosis group, except for macrophages which showed significantly higher scores in the high-risk poor prognosis group (Fig. 8I). Additionally, we observed significant differences in the immune function scores of chemokine receptor (CCR), checkpoint, cytolytic_activity, inflammation-promoting, para-inflammation, T cell co-stimulation, and type I IFN_response between the two groups (Fig. 8J). Subsequently, we analyzed the expression levels and differences in immune checkpoint-related genes between the high-risk poor prognosis and low-risk good prognosis groups. As a result, all checkpoint genes except NRP1, PDCD1LG2, CD44, and CD276 were expressed at higher levels in the low-risk good prognosis group compared to the high-risk poor prognosis group (Fig. 8K). The aforementioned findings may offer innovative targets for the immunotherapy of patients with HNSCC.

### Drug sensitivity in the ERS-related lncRNA prognostic signature

**Figure 9:**
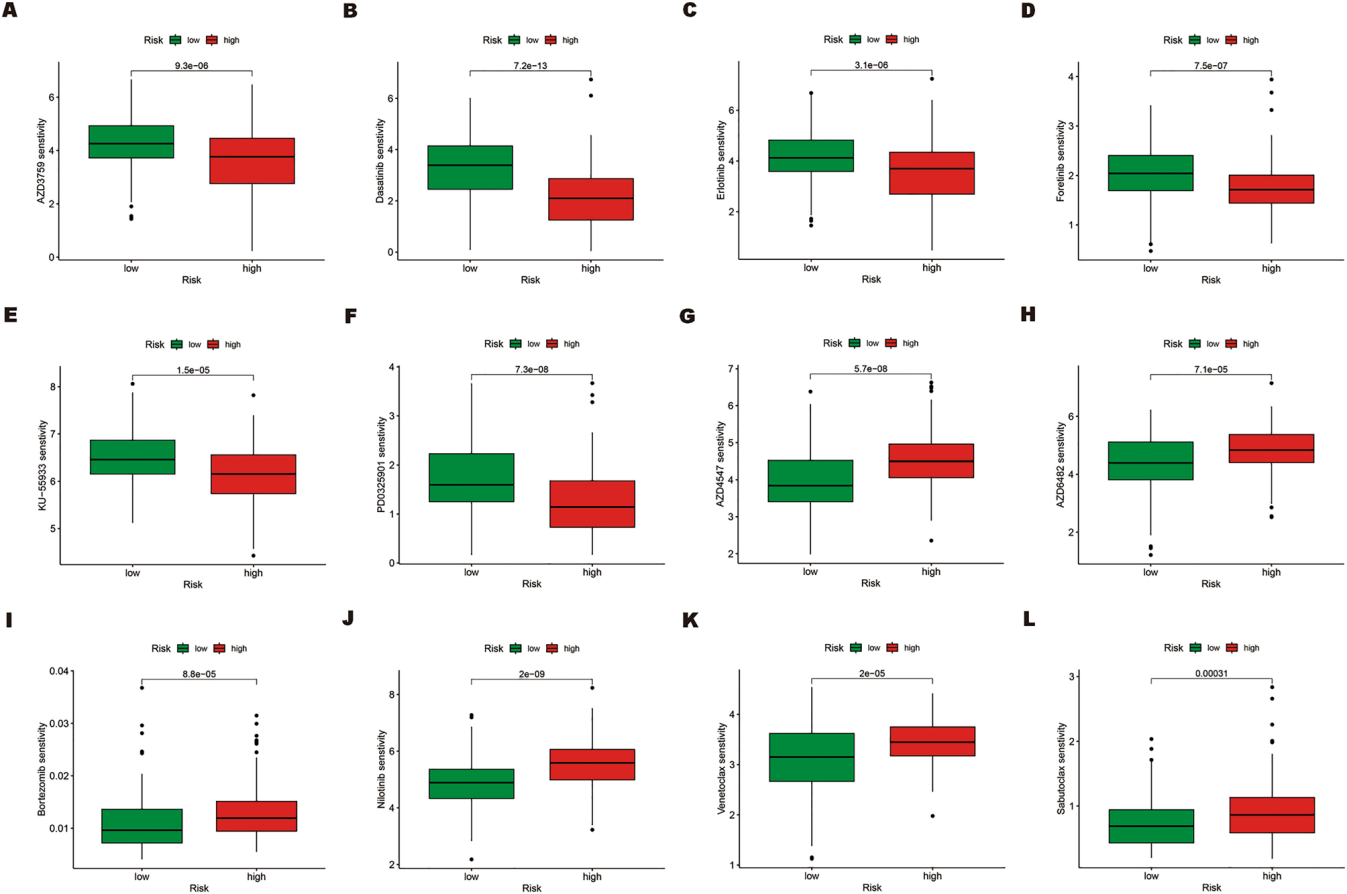
Comparison of the IC50 of different drugs in the high-risk poor prognosis and low-risk good prognosis groups. (**A–L**) The IC50 of AZD3759, Dasatinib, Erlotinib, Foretinib, KU-55933, PD0325901, AZD4547, AZD6482, Bortezomib, Nilotinib, Vorinostat, and Sabutoclax in the high-risk poor prognosis and low-risk good prognosis groups. IC50, the half-maximal inhibitory concentration. *, p < 0.05; **, p < 0.01; ***, p < 0.001.

At present, there is still a significant gap in the drug treatment of HNSCC, which forces us to find potential therapeutic drugs for this condition. Drug sensitivity analysis is essential to achieve personalized treatment for cancer patients and promote the development of precision medicine. For this purpose, we calculated the IC50 of different drugs in the high-risk poor prognosis and low-risk good prognosis groups to screen out drugs with differential sensitivity between the two groups. The findings demonstrated that, in comparison to the low-risk good prognosis group, the high-risk poor prognosis patients were more sensitive to AZD3759 (EGFR inhibitor), Dasatinib (SRC inhibitor), Erlotinib (EGFR inhibitor), Foretinib (VEGFR/HGFR inhibitor), KU-55933 (ATM Kinase Inhibitor), and PD0325901 (MEK/ERK pathway inhibitor), while they were more resistant to AZD4547 (FGFR inhibitor), AZD6482 (PI3Kβ inhibitor), Bortezomib (proteasome inhibitor), Nilotinib (Bcr-abl inhibitor), Venetoclax (Bcl-2 inhibitor), and Sabutoclax (Bcl-2 inhibitor) than low-risk patients(Fig. 9A–L). These findings will also facilitate the formulation of distinct therapeutic strategies for high-risk poor prognosis and low-risk good prognosis cohorts, thereby contributing to the advancement of precision medicine in HNSCC.

### Consensus clustering determined three ERS-related clusters of HNSCC

**Figure 10:**
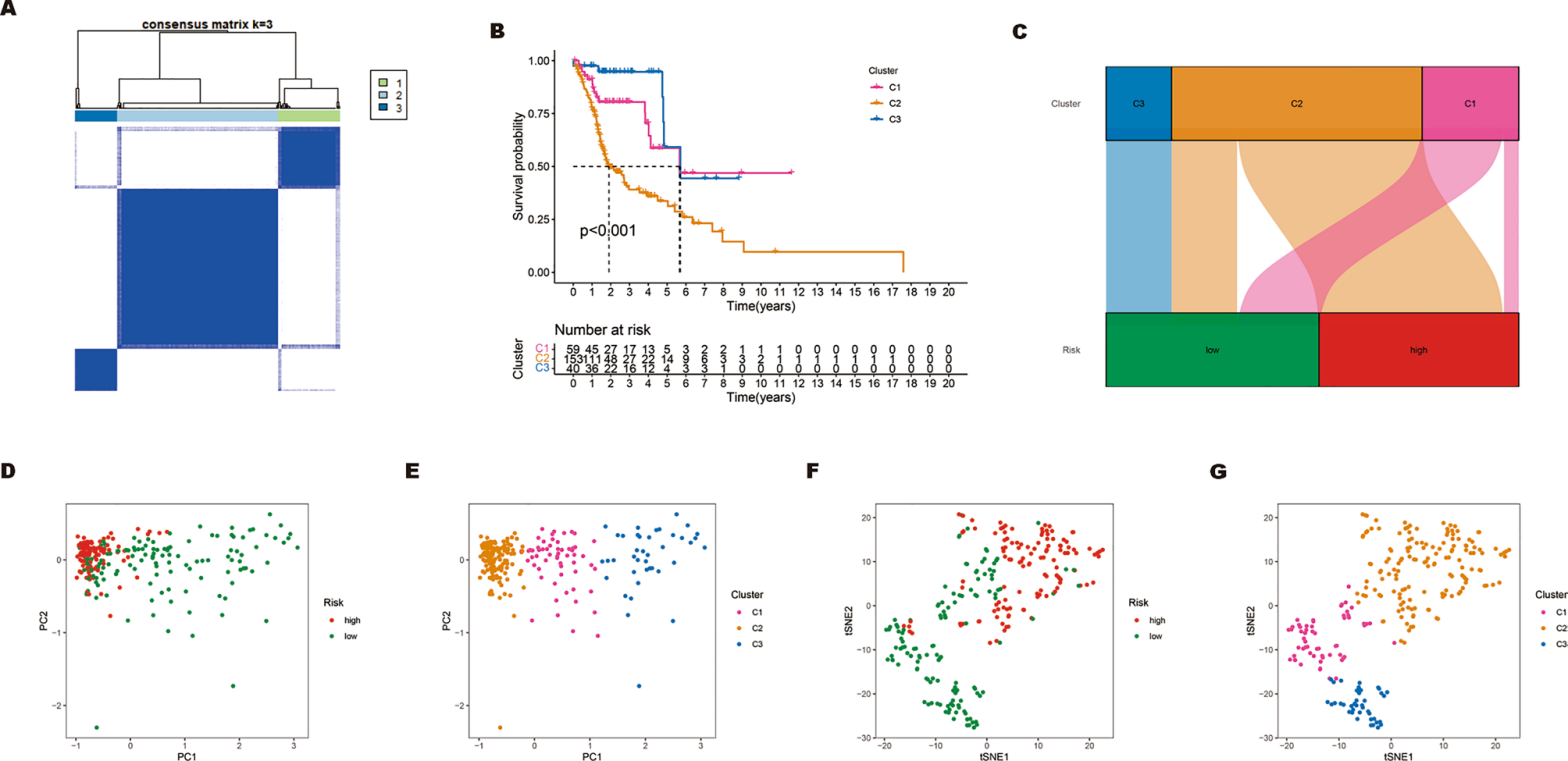
Survival, PCA, and tSNE analysis of three different subgroups of HNSCC divided by consensus clustering. (**A**) Consensus matrix with optimal k = 3. (**B**) Kaplan–Meier survival curves of HNSCC patients’ OS among the three different subgroups. (**C**) The Sankey diagram shows the connection degree between the three different subgroups and risk score. (**D**) Principal component analysis between the high-risk poor prognosis and low-risk good prognosis groups. (**E**) Principal component analysis among the three different subgroups. (**F**) tSNE analysis between the high-risk poor prognosis and low-risk good prognosis groups. (**G**) tSNE analysis among the three different subgroups.

**Figure 11:**
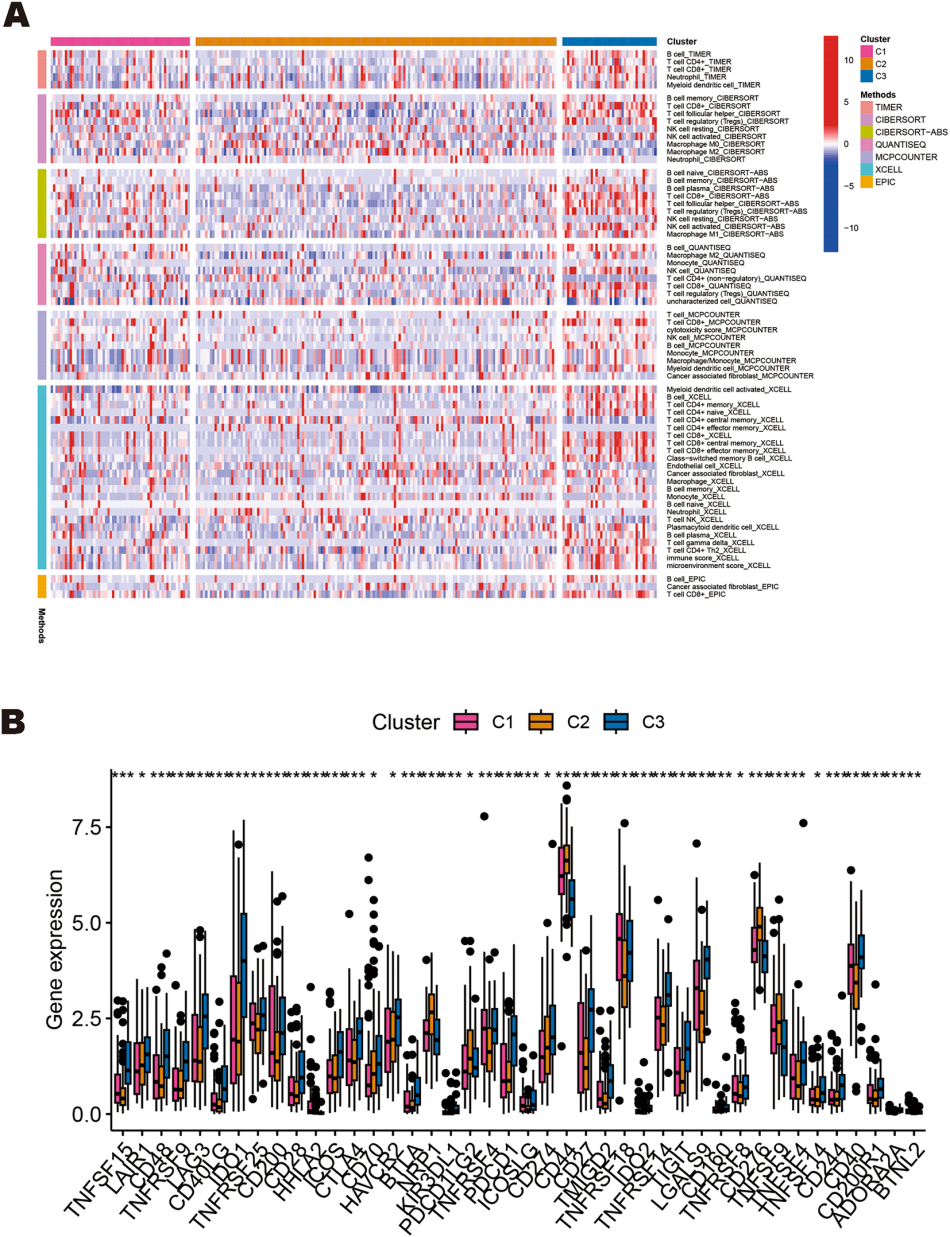
The immune cell infiltration landscape and the expression of immune checkpoint-related genes among the three different subgroups of HNSCC. (**A**) Heatmap for immune cell infiltration landscape based on the CIBERSORT, CIBERSORT-ABS, QUANTISEQ, XCELL, MCP-counter, EPIC, and TIMER algorithms among the three different subgroups. (**B**) The expression levels of immune checkpoint-related genes among the three different subgroups.

**Figure 12:**
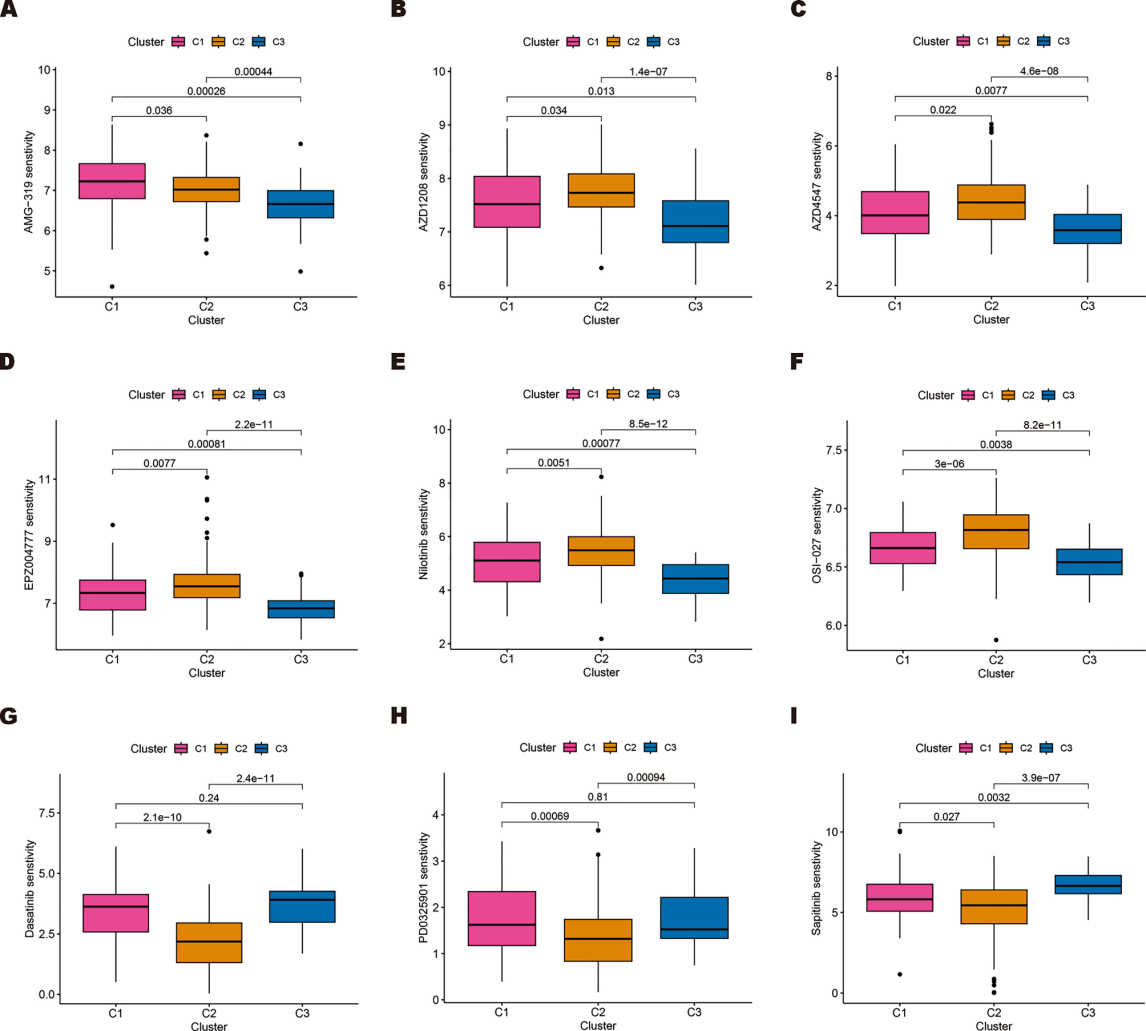
Drug sensitivity analysis of potential therapeutic drug among the three subgroups. (**A–I**) Drug sensitivity (IC50) analysis of AMG-319, AZD1208, AZD4547, EPZ004777, Nilotinib, OSI-027, Dasatinib, PD0325901, and Sapitinib in three subgroups.

To identify and compare the molecular subtypes of HNSCC, we performed a consensus cluster analysis of the seven ERS-related lncRNAs. Based on the heatmaps of the consensus matrix under different numbers of clusters (k), we conclude that k = 3 is the optimal k value to maintain the stability of clusters. TCGA-HNSCC samples were divided into three clusters: cluster 1 (n = 59), cluster 2 (n = 153), and cluster 3 (n = 40) (Fig. 10A). Next, we compared differences in the OS, immune cell infiltration level, immune checkpoint genes expression, and drug sensitivity among the three clusters. From Fig. 10B, it can be seen that there are differences in the OS of patients among the three subgroups, and patients in cluster 2 had a worse prognosis for OS compared to those in cluster 1 and cluster 3 (*p* < 0.001). The correspondence between sample subgroups and patient risk was that cluster 3 was all low-risk patients, and cluster 1 and cluster 2 were mostly high-risk poor prognosis patients (Fig. 10C). The results of PCA and t-SNE analysis indicate significant dimensional differences among different subgroups, as well as different dimensions between high-risk poor prognosis and low-risk good prognosis groups (Fig. 10D–G). Subsequently, the heatmap was plotted to show the infiltration level of immune cells in three subgroups, indicating that cluster 3 has the highest level of immune cell infiltration (Fig. 11A). In addition, we evaluated immune checkpoint gene expression in three clusters, and the results showed that NRP1, PDCD1LG2, CD44, CD276, and TNFSF9 were highly expressed in cluster 2 (Fig. 11B). Finally, we identified 84 drugs with differential sensitivities in different HNSCC subgroups by comparing the sensitivities of patients in three subgroups to 197 drugs. From Fig. 12A–I, we found that HNSCC patients in cluster 3 were sensitive to most drugs, including AMG-319 (PI3K inhibitor), AZD1208 (PIM inhibitor), AZD4547 (FGFR inhibitor), EPZ004777 (DOT1L inhibitor), Nilotinib (Bcr-abl inhibitor), and OSI-027 (mTOR inhibitor). While those in cluster 2 were more sensitive to Dasatinib (Bcr-abl inhibitor), PD0325901 (MEK/ERK pathway inhibitor), and Sapitinib (EGFR inhibitor). The above analysis suggests that patients with HNSCC in cluster 3 may have a more favorable prognosis and therapeutic response compared to other subgroups.

### Validation of seven ERS-related lncRNAs in HNSCC

**Figure 13:**
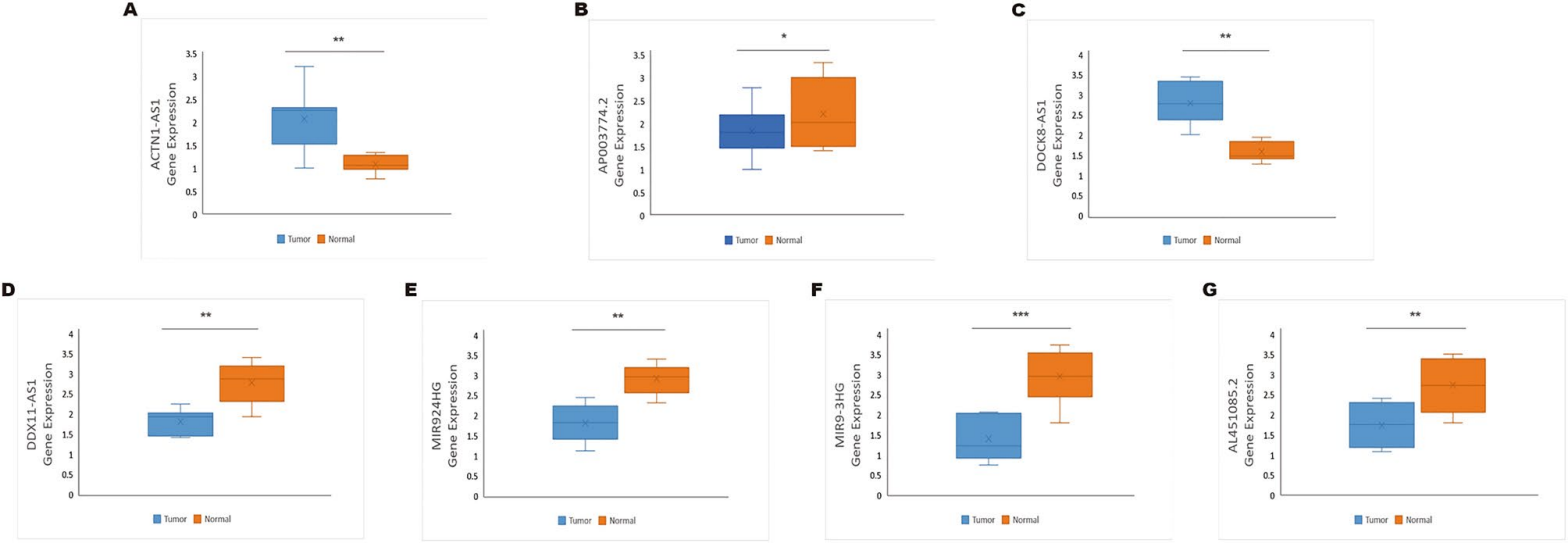
The expression level of the seven ERS-related lncRNAs in HNSCC tissues and normal tissues. (**A–G**) The expression of ACTN1-AS1, AP003774.2, DOCK8-AS1, DDX11-AS1, MIR924HG, MIR9-3HG, and AL451085.2 in HNSCC tissues and normal tissues by qRT-PCR. *P < 0.05, **P < 0.01, and ***P < 0.001.

To further investigate the expression of these seven ERS-related lncRNAs in HNSCC, we utilized qRT-PCR to compare their expression levels between HNSCC tissues and normal tissues. qRT-PCR showed that the expression of AP003774.2, DDX11-AS1, MIR924HG, MIR9-3HG, and AL451085.2 were downregulated, while ACTN1-AS1 and DOCK8-AS1 were upregulated in HNSCC tissues compared to paired normal tissues (Fig. 13A–G). The findings are in line with the aforementioned outcomes of our bioinformatics analysis.

## Discussion

HNSCC has an insidious onset and a complex anatomical location, which makes early diagnosis difficult. Additionally, 70–80% of HNSCC patients are diagnosed at an advanced stage. What's worse, they remain susceptible to recurrence and metastasis even after undergoing surgery, radiotherapy, or chemotherapy^37^. Despite the emergence of the immune era and increasing utilization of immunotherapy, a significant proportion of patients still exhibit primary resistance to ICIs and do not derive clinical benefit, resulting in a dismal prognosis^38^. Therefore, we should focus on exploring effective markers for early diagnosis of HNSCC and potential therapeutic targets for HNSCC more thoroughly. ERS is an important cellular defense mechanism that regulates protein folding and maintains endoplasmic reticulum homeostasis. It is associated with various processes in tumor cell biology, including angiogenesis, treatment resistance, invasion, and inflammation^39^. The involvement of lncRNAs in diverse cancer types has been well-documented, highlighting their pivotal roles in oncogenesis^12^. Therefore, in this study, we mainly investigated the potential of ERS-related lncRNAs as prognostic or diagnostic markers and therapeutic targets for HNSCC through bioinformatics analysis.

In this study, we identified ERS-related lncRNAs associated with prognosis based on ERS-related genes and HNSCC patient samples from the TCGA database. Subsequently, we developed a novel prognostic model consisting of seven ERS-related lncRNAs. Previous studies have also constructed ERS-related models in various tumors, such as lung adenocarcinoma, breast cancer, hepatocellular carcinoma, and ovarian cancer^40–43^. However, the prognostic potential of ERS-related lncRNAs in HNSCC remains largely unexplored. Here, we found that seven ERS-related lncRNAs (ACTN1-AS1, AP003774.2, DOCK8-AS1, DDX11-AS1, MIR924HG, MIR9-3HG, and AL451085.2) were differentially expressed between tumor and normal tissues and associated with the OS of HNSCC patients. Furthermore, we observed downregulation of the expression of AP003774.2, DDX11-AS1, MIR924HG, MIR9-3HG, and AL451085.2 in HNSCC tissues compared to normal tissues, while ACTN1-AS1 and DOCK8-AS1 were upregulated. Currently, research on some lncRNAs in our model is still lacking or only in its early stages, while previous studies have shown that certain lncRNAs are closely related to tumorigenesis and prognosis.

DDX11-AS1, as a typical oncogene, may promote the growth and metastasis of cancer cells by regulating gene expression related to cell cycle, DNA repair, and transcriptional regulation. Its overexpression is often associated with poor clinicopathological features; therefore, it could potentially be developed as a tumor marker for diagnosis and prognosis^44^. Studies have found that MIR9-3HG is associated with the survival time of HNSCC, and a higher expression of MIR9-3HG is correlated with a better prognosis compared to the low-expression group^45^. A recent study has shown that AL451085.2, a ferroptosis-related lncRNA, has a favorable prognostic effect on patients with HNSCC^46^. Additionally, studies have also found a significant decrease in the expression of DOCK8-AS1 in renal cancer patients, and those with low DOCK8-AS1 expression have a poor prognosis^47^. However, there is a lack of studies investigating the relationship between ACTN1-AS1, AP003774.2, MIR924HG, and tumors in HNSCC patients. Therefore, further research is warranted to elucidate the potential mechanism underlying these lncRNAs.

In this study, we not only constructed a prognostic model for ERS-related lncRNAs but also verified its predictive ability. We divided 252 HNSCC patients in the training and testing sets into high-risk poor prognosis and low-risk good prognosis groups based on the median threshold of their risk scores. We observed that the low-risk good prognosis group had higher OS. Furthermore, this model could effectively predict the survival of HNSCC patients independently of other clinical factors. To further reveal the potential mechanisms of our ERS-related model associated with HNSCC prognosis, GO, KEGG, and GSEA were employed in this study. GO enrichment analysis revealed that the differentially expressed genes were mainly enriched in immune-related functions and pathways, indicating that the difference in prognosis between the high-risk poor prognosis and low-risk good prognosis groups may be attributed to differences in immune cell infiltration and function. Remarkably, we observed a significant enrichment of differentially expressed genes in specific signal transduction pathways and molecules through KEGG analysis. These pathways encompass cytokine-cytokine receptor interaction, JAK-STAT signaling pathway, and cell adhesion molecules, indicating a potential correlation between our ERS-related model and the growth, proliferation, migration, and invasion of cancer cells.

Studies have demonstrated that tumors with a high TMB tend to exhibit enhanced sensitivity toward immunotherapy and have a more favorable prognosis^48^. Our results showed that the mutation rate of the TP53 gene was high in both high-risk poor prognosis and low-risk good prognosis groups. The group with a high mutation rate presented a better prognosis, while HNSCC patients with a combination of high-risk and low-TMB had worse OS. The aforementioned results can assist us in identifying patients who may benefit from targeted therapy and guide the development of personalized treatment plans. The abundance, variety, and activation status of immune cell infiltration within the tumor microenvironment have been demonstrated to play a pivotal role in tumorigenesis and can impact the clinical prognosis of cancer patients^49^. The majority of studies indicate that increased immune cell infiltration is generally linked to a more favorable prognosis^50^. Our study revealed a significant negative association between risk scores and the majority of infiltrating immune cells. HNSCC patients with high levels of infiltration of B cell, Macrophage M2, T cell CD4+ naive, T cell CD8+, T cell follicular helper, and T cell regulatory T cells (Tregs) showed higher OS. Using ssGSEA, we explored the immune status of high-risk poor prognosis and low-risk good prognosis groups and observed that the low-risk good prognosis had more immune cell infiltrations and stronger anti-tumor immunity. Based on these findings, we hypothesized that poor prognosis in HNSCC patients from the high-risk poor prognosis group may be due to low immune cell infiltration and weak anti-tumor immune function. Additionally, we compared the expression levels of immune checkpoint-related genes among different groups and found that NRP1, PDCD1LG2, CD44, and CD276 were highly expressed in the high-risk poor prognosis group. These genes are expected to become new targets for immunotherapy.

With the deepening of research, drug therapy for HNSCC has made significant progress^51^. However, there is still a considerable distance to cover in achieving effective and precise treatment. Based on our ERS-related lncRNAs prognostic model, we found that high-risk poor prognosis patients were more sensitive to AZD3759, Dasatinib, Erlotinib, Foretinib, KU-55933, and PD0325901 compared to the low-risk good prognosis group. Our results can help improve treatment outcomes for HNSCC patients and reduce unnecessary drug side effects. Finally, we stratified HNSCC patients into three subgroups to investigate the similarities and differences among tumor subtypes. Cluster 3, comprising solely of low-risk good prognosis patients, exhibited the highest infiltration of immune cells and expression of immune checkpoint molecules. It is plausible that HNSCC patients in cluster 3 may have a more favorable prognosis and response to immunotherapy. Conversely, patients in cluster 2 had a poorer prognosis. Furthermore, we observed that HNSCC patients in cluster 3 were responsive to most drugs including AMG-319, AZD1208, AZD4547, EPZ004777, Nilotinib, and OSI-027. Collectively, these findings suggest that tumor subtypes can inform treatment options and enhance patient outcomes for HNSCC.

## Conclusion

A robust predictive model has been constructed based on seven ERS-related lncRNAs. This prognostic model shows promise as a tool for risk stratification, survival prediction, and assessment of immune cell infiltration status, which has the potential to facilitate personalized and accurate therapy for patients with HNSCC.

## Data Availability

The original contributions presented in the study are included in this article. Further inquiries can be directed to the corresponding authors.
